# Syndrome of Inappropriate Antidiuretic Hormone Secretion and Cerebral/Renal Salt Wasting Syndrome: Similarities and Differences

**DOI:** 10.3389/fped.2014.00146

**Published:** 2015-01-22

**Authors:** Ji Young Oh, Jae Il Shin

**Affiliations:** ^1^Department of Pediatrics, Severance Children’s Hospital, Yonsei University College of Medicine, Seoul, South Korea

**Keywords:** hyponatremia, syndrome of inappropriate antidiuretic hormone secretion, cerebral/renal salt wasting syndrome, volume status, fractional excretion of urate

## Abstract

Hyponatremia (sodium levels of <135 mEq/L) is one of the most common electrolyte imbalances in clinical practice, especially in patients with neurologic diseases. Hyponatremia can cause cerebral edema and brain herniation; therefore, prompt diagnosis and proper treatment is important in preventing morbidity and mortality. Among various causes of hyponatremia, diagnosing syndrome of inappropriate antidiuretic hormone secretion (SIADH) and cerebral/renal salt wasting syndrome (C/RSW) is difficult due to many similarities. SIADH is caused by excess of renal water reabsorption through inappropriate antidiuretic hormone secretion, and fluid restriction is the treatment of choice. On the other hand, C/RSW is caused by natriuresis, which is followed by volume depletion and negative sodium balance and replacement of water and sodium is the mainstay of treatment. Determinating volume status in hyponatremic patients is the key point in differential between SIADH and C/RSW. However, in most situations, differential diagnosis of these two diseases is difficult because they overlap in many clinical and laboratory aspects, especially to assess differences in volume status of these patients. Although distinction between the SIADH and C/RSW is difficult, improvement of hypouricemia and an increased fractional excretion of uric acid after the correction of hyponatremia in SIADH, not in C/RSW, may be one of the helpful points in discriminating the two diseases. In this review, we compare these two diseases regarding the pathophysiologic mechanisms, diagnosis, and therapeutic point of view.

## Introduction

Hyponatremia, defined by sodium levels of <135 mEq/L, is one of the common electrolyte imbalances in clinical practice, especially among patients with neurologic diseases (1). Prevalence of hyponatremia is approximately 15–30% in hospitalized patients, both children and adults, and 1–8% in outpatient clinic setting (2). Acute hypoosmolality caused by serum sodium levels of 110–120 mEq/L can cause cerebral edema and herniation (3–5). After this osmotic insult, the adaptation mechanism of losing the electrolytes and organic osmolytes in brain tissue facilitates restoration of the brain volume (6, 7). This mechanism is known as “‘osmoregulation” (8, 9). However, correcting sodium levels too fast for the brain tissue to adapt, especially in patients with more vulnerable brain tissue due to neurologic disorders, can impair this osmoregulatory mechanism (9). Therefore, patients in these situations present brain deterioration that is more serious and have show increased mortality rates (4, 9). Thus, rapid diagnosis of hyponatremia is important in preventing morbidity and mortality in patient management, especially in neurologic intensive care settings.

Among various causes of hyponatremia, the diagnoses of the syndrome of inappropriate antidiuretic hormone secretion (SIADH) and cerebral/renal salt wasting syndrome (C/RSW) are still confusing due to many similarities. According to one retrospective study of electrolyte disturbances in 195 children with acute central nervous system (CNS) diseases, 20 (10.3%) children showed hyponatremia, 7 of them were diagnosed with SIADH, and the other 9 with cerebral salt wasting syndrome (CSW) (1).

Although it may be difficult to distinguish between the two diseases, it is important to do so since the treatment of the two diseases differ in many aspects (10). In this review, we would like to compare these two diseases, which have many differences related to the pathophysiologic mechanisms, diagnosis, and therapeutic points of view despite the similarities.

## Definitions and Pathophysiologic Mechanisms of SIADH

SIADH is a disease categorized as hypo-osmolar hyponatremia; small amount of volume expansion is caused by excess of renal water reabsorption through inappropriate antidiuretic hormone (ADH) secretion (11). Before the diagnosis of SIADH, other diseases such as dysfunctions of heart, liver and kidneys, adrenal insufficiency, and hypothyroidism, should be excluded. Excessive ADH secretion causes water retention by increasing water permeability in the renal collecting duct (12). Consequently, the increased glomerular filtration rate (GFR) due to the volume expansion and vasodilating effect of increased circulating atrial/brain natriuretic peptides can increase sodium excretion but there is also decreased tubular transport of sodium due to unknown mechanism (12, 13). In addition, natriuretic peptides are released primarily due to vasopressin stimulation (14) and, secondarily due to overfilling of the plasma volume by homeostatic mechanisms (15). Increased pressure in the glomerular capillaries and decreased plasma renin and aldosterone secretion can lead to natriuresis by increasing GFR and blocking sodium reabsorption in the collecting duct (14–16).

## Definitions and Pathophysiologic Mechanisms of C/RSW

C/RSW is another important disease category causing hyponatremia, characterized by volume depletion and negative sodium balance. Peters et al. first described C/RSW in a report of three patients with neurogenic diseases and hyponatremia (17). Following the original description of SIADH by Schwartz et al. in 1957, the term C/RSW had not been used for 20 years (12). Then, in 1981, Nelson et al. studied hyponatremia in patients undergoing neurosurgery, and re-established interest in C/RSW (18). In that journal, Nelson et al. determined blood volume by gold standard radioisotope dilution methods and demonstrated a large number of neurosurgical patients to be volume depleted with high urinary sodium excretion that were very consistent with salt wasting (18). Although C/RSW has also been considered to be caused by sequential complications of CNS diseases (19–21), some reports show that it could occur in patients without CNS diseases; therefore, it is recently referred to as renal salt wasting (RSW) (22, 23). As a result of these studies, we agree with changing CSW to RSW because CSW would not be considered in patients without cerebral disease, the prevalence of which has yet to be determined.

The pathophysiology and mechanisms of C/RSW are not completely understood, nevertheless, there is general agreement that the kidneys are unable to conserve sodium leading to variable degrees of reduction in extracellular volume (ECV) depending the extent of the defect in sodium transport and salt intake. In addition, the patient must also have a normally functioning kidney and hypothalamic–pituitary–adrenal axis in order to be diagnosed as C/RSW (17).

This syndrome could be explained with two main hypotheses (4, 24, 25): decreased sympathetic outflow to the kidney and amplification of the circulating natriuretic peptide, particularly brain natriuretic peptide (24–26). Sympathetic tone plays an important role in the kidney in sodium and water absorption at proximal tubules and releasing renin at the juxtaglomerular epithelioid cells (25, 27). Therefore, theoretically, renin–aldosterone levels are not increased due to reduced sympathetic tone in the kidney despite the volume depletion in C/RSW patients (16), but renin–aldosterone levels have been reported to be high in salt wasting (22). The patient with C/RSW initially loses sodium but eventually reaches a steady state where sodium excretion matches sodium intake, and urinary sodium can be low despite having salt wasting. In addition, increased natriuretic peptides induce natriuresis by increasing GFR and preventing sodium reabsorption in the collecting duct (28, 29). Also, natriuretic peptides could inhibit the action of aldosterone, antagonize the arginine vasopressin (AVP) effects and reduce sympathetic outflow (16, 24, 26). However, Maesaka et al. reported that natriuretic peptides were found to be low in a volume depleted patient with RSW and suggested their contribution to RSW is not supported by their modest effect on sodium and other solute transport in the proximal tubules (22, 23).

If ECV is decreased due to C/RSW, the baroreceptors are triggered and ADH is secreted to restore intravascular volume. The stimulus for ADH secretion by the reduced ECV is more potent than the osmolar effect on ADH secretion, so the patient remains hyponatremic despite the hypoosmolality. This defines the appropriateness of ADH secretion in C/RSW and why saline can remove the volume stimulus for ADH secretion and allow the hypoosmolality to inhibit ADH secretion, increase free water excretion and correct the hyponatremia.

## SIADH and C/RSW: Similarities and Differences

Distinguishing between SIADH and C/RSW is important since the proper treatment for each disease is different, and erroneous diagnosis could endanger the patient. However, it is difficult to distinguish the two diseases because many clinical symptoms and laboratory findings overlap. Hyponatremia is a common finding of both SIADH and C/RSW, but the most important difference between the two diseases is the patient’s volume status. SIADH tends to be euvolemic or slightly hypervolemic, whereas C/RSW tends to be hypovolemic (13, 24). Patients with SIADH do not present overt hypervolemic signs clinically, since one-third of the retention fluid remains in the extracellular space (13). Therefore, it is difficult to distinguish between the two diseases with only clinical symptoms and physical examinations (e.g., edema or neck vein distension), measurements of blood pressure, or pulse rate according to posture change. There have been some methods for the direct estimation of circulating blood volume by central venous catheter (measurement of central venous pressure) or Swan–Ganz catheter (measurement of pulmonary capillary wedge pressure) in patients without cardiac or pulmonary diseases (4, 13, 30). However, CVP determinations do not accurately determine the volume status of patients when compared to gold standard radioisotope dilution methods, and there is no exact and easily applicable method, which can be used to define volume status in children.

In addition, urine electrolytes can be used to evaluate volume status (31, 32). Generally, it is known to be helpful diagnosing SIADH when the patient exhibits urinary sodium and chloride excretion increased more than 30 mmol/L and fractional excretions of sodium and chloride more than 0.5% (33). In SIADH patients with water retention, hypertension or catecholamine-induced vasoconstriction can also induce aggressive natriuresis temporarily, and therefore measuring mass balance of urinary electrolytes over a few days may be more helpful to make a decision (24, 34). Unfortunately, there is no gold standard cut-off value of urinary sodium excretion to distinguish the two diseases.

Although laboratory findings such as elevated hematocrit, plasma blood urea nitrogen (BUN), or albumin level might reflect low effective blood volume in C/RSW (13, 33, 35), it may be difficult to evaluate volume status accurately. Serum uric acid levels are generally decreased and fractional excretions of uric acid (FEUrate) are increased both in SIADH and C/RSW (12, 33, 36), although the mechanisms are not clear. Normal value of the FEUrate in euvolemic patients is about 10% and is reduced (usually <5%) in hypovolemic patients (37, 38). In most SIADH and C/RSW patients, the level of FEUrate is more than 10%. Maesaka et al. noted that hypouricemia and an increased FEUrate improve in SIADH, but not in C/RSW after the correction of hyponatremia (23, 37, 39). This might be associated with impaired sodium-urate-co-transporter in the proximal tubule itself in C/RSW. The FEUrate is suggested that it could be used in the algorithm to distinguish SIADH from C/RSW (22, 23). However, an exact value and pathophysiologic mechanisms of decreased serum uric acid level in pediatric patients remain elusive.

Although not tested in children, one recent report demonstrated that bioimpedance spectroscopy showed a higher level of agreement with clinical body fluid estimation than physical examination in adults with hyponatremia, suggesting that bioimpedance spectroscopy could replace physical examination for estimating body fluid status in hyponatremia (40). Because bioimpedance spectroscopy is also used to measure hydration status in children receiving hemodialysis (41), it would be interesting to study if this method could be useful in determining the volume status in children with hyponatremia in the future. However, special attention should be paid utilization of bioimpedance is most effective when volumes are determined at two time intervals where the changes noted between both points are made.

## Treatment

Because hyponatremia is poorly tolerated in patients with C/RSW diseases, and even a small decrease in serum sodium can aggravate vasogenic cerebral edema (42), it should be rapidly recognized and managed promptly. Vasogenic edema is accumulating the protein-rich fluid to the extracellular space resulted from destruction of the blood–brain barrier due to increased vascular permeability (43–46).

Three percent NaCl should be administered for acute symptomatic hyponatremia. Excessive correction of chronic hyponatremia should be avoided as it can result in osmotic demyelination (10, 47). Though it is rare, osmotic demyelination is a serious and fatal complication that can cause aggressive neurologic deficit such as seizure, quadriplegia, coma, and even death (6, 47, 48).

Simple fluid restriction is recommended for the treatment of hyponatremia in uncomplicated SIADH (11, 49). Isotonic saline is not usually effective in increasing serum sodium levels in SIADH, because water will be retained and sodium will be excreted in urine, causing possible aggravation of hyponatremia. Hypertonic saline can increase serum sodium levels, but the response will partially dissipate over time and therefore, oral salt tablets with loop diuretics can be considered as a treatment of hyponatremia caused by SIADH (50). Vasopressin receptor antagonists (vaptans) emerged as a new class of drugs for the treatment of euvolemic and hypervolemic hyponatremia such as SIADH in adults (51). Although there have been scarce studies of data research on the use of vaptans in children (52, 53), Jones et al. showed that intravenous conivaptan was effective for increasing serum sodium levels and might be a potential adjuvant to enhance diuresis in children with cardiac disease (52) and Horibata et al. reported oral tolvaptan improved the condition of 6-year-old boy with the loop diuretic-resistant congestive cardiac failure without hypernatremia, deterioration of vital signs, and significant complications (53). The common side effects of vaptans are thirst and increased urination (2, 54). Recently, one case report of over-correction of hyponatremia with development of severe hypernatremia and demyelination with usage of vaptan was published (2, 55). In addition, one report was reported that long term usage of tolvaptan was resulted in severe hepatotoxicity in a patient with autosomal dominant polycystic kidney disease (2, 56). Some reports suggested contraindications of vaptan (57, 58). Vaptan therapy is absolutely contraindicated for patients in hypovolemic hyponatremia, because these patients already experienced loss of water and sodium that exceeded hemodynamic compensation and renal function (57, 58). Therefore, safety concerns and clinical trials regarding vaptan use in children with SIADH will be necessary in the future.

The treatment purpose of C/RSW is to restore decreased serum sodium levels and intravascular volume due to natriuresis and diuresis and normal saline is frequently used as an initial fluid (4, 30, 59). If the patient with salt wasting becomes euvolemic with saline, the hyponatremia should be corrected in a short period of time. If it does not, there should be a question of the accuracy of the diagnosis. At the start of the treatment, it might be safer to use hypertonic saline if the patient has symptomatic hyponatremia and then switch to isotonic saline, which should eliminate the more potent volume stimulus for ADH secretion. To make strengthen the diagnosis of salt wasting, testing for excretion of diluted urines may be useful. Once euvolemia is achieved, the degree of hyponatremia should be re-evaluated. If serum sodium levels are severely decreased (<125 mEq/L) or a large volume of intravenous fluids is required to maintain euvolemia, intravenous hypertonic saline can also be used (4). A hypertonic 3% NaCl infusion can be titrated with a 0.9% NaCl infusion to obtain the desired sodium concentration to maintain appropriate serum sodium (44, 60), or alternatively, mineralocorticoids can be used to augment both serum sodium concentration and intravascular volume (61–64). Oral salt supplementation can be used in a stable patient with CNS disease and C/RSW.

## Conclusion

Because SIADH and C/RSW have many similarities, differential diagnosis is very difficult in patients with hyponatremia, especially in those with CNS diseases. Nevertheless, it is also important to distinguish between SIADH and C/RSW since the treatment of them differ in many aspects. Volume status of the patients, though it may be difficult to evaluate in the clinical setting, serum uric acid levels and FEUrate after correcting hyponatremia, and the degree of fractional excretions of sodium may be useful in differentiating between the two diseases. Therefore, further studies regarding pathophysiologic mechanisms of diseases and development of diagnostic tools to determine exact volume status is necessary in the future.

## Conflict of Interest Statement

The authors declare that the research was conducted in the absence of any commercial or financial relationships that could be construed as a potential conflict of interest.
